# New Python-based methods for data processing

**DOI:** 10.1107/S0907444913000863

**Published:** 2013-06-18

**Authors:** Nicholas K. Sauter, Johan Hattne, Ralf W. Grosse-Kunstleve, Nathaniel Echols

**Affiliations:** a Physical Biosciences Division, Lawrence Berkeley National Laboratory, 1 Cyclotron Road, Berkeley, CA 94720, USA

**Keywords:** data processing, reusable code, multiprocessing, *cctbx*

## Abstract

Current pixel-array detectors produce diffraction images at extreme data rates (of up to 2 TB h^−1^) that make severe demands on computational resources. New multiprocessing frameworks are required to achieve rapid data analysis, as it is important to be able to inspect the data quickly in order to guide the experiment in real time. By utilizing readily available web-serving tools that interact with the Python scripting language, it was possible to implement a high-throughput Bragg-spot analyzer (*cctbx.spotfinder*) that is presently in use at numerous synchrotron-radiation beamlines. Similarly, Python interoperability enabled the production of a new data-reduction package (*cctbx.xfel*) for serial femto­second crystallography experiments at the Linac Coherent Light Source (LCLS). Future data-reduction efforts will need to focus on specialized problems such as the treatment of diffraction spots on interleaved lattices arising from multi-crystal specimens. In these challenging cases, accurate modeling of close-lying Bragg spots could benefit from the high-performance computing capabilities of graphics-processing units.

## Abstract 2

The *Computational Crystallography Toolbox* (*cctbx*) is a flexible software platform that has been used to develop high-throughput crystal-screening tools for both synchrotron sources and X-ray free-electron lasers. Plans for data-processing and visualization applications are discussed, and the benefits and limitations of using graphics-processing units are evaluated.

## 1. Introduction

It is widely recognized that modular and reusable code speeds up software development. In crystallography, the collaborative efforts embodied in the *CCP*4 software suite have been greatly facilitated by the availability of libraries (Winn *et al.*, 2002 ▶, 2011 ▶) that provide common file formats for describing atomic structures, structure factors and electron-density maps. Two additional features of software design have also been important: object-oriented programming (Grosse-Kunstleve *et al.*, 2002 ▶; Cowtan, 2003 ▶), which allows computational problems to be attacked at a high level of abstraction, and scripting, which enables the rapid testing of new ideas. Modules that implement these principles, such as those in the *Computational Crystallography Toolbox* (*cctbx*; Grosse-Kunstleve *et al.*, 2002 ▶; Bourhis *et al.*, 2007 ▶), have been used as efficient building blocks for numerous crystallographic applications, including small-molecule (Dolomanov *et al.*, 2009 ▶) and macromolecule (Adams *et al.*, 2010 ▶) structure solution, indexing (Sauter *et al.*, 2004 ▶) and scaling (Evans, 2006 ▶), and data-processing workflows (Winter, 2010 ▶). The ability to customize scripted tools for use in new contexts, and the capacity of various software toolboxes to work together, are key ingredients in developing new programs that support the crystallography experiment as it evolves over time.

X-ray crystallographic data collection has profoundly changed in recent years with the introduction of fast area detectors based either on pixel-array area-detector (PAD) or p–n junction charge-coupled device (pnCCD) technologies. These detectors enable framing rates ranging from 25 Hz at synchrotron sources (Eikenberry *et al.*, 2003 ▶) to 120 Hz at free-electron lasers (Strüder *et al.*, 2010 ▶; Philipp *et al.*, 2010 ▶). With such high framing rates, the inspection and analysis of data has emerged as a potential bottleneck, requiring new multiprocessing approaches for timely data processing. One important element, of course, is the allocation of sufficient computing hardware, but software design is also paramount for increasing efficiency. New software is needed to correctly model the physical properties unique to these detectors, such as neighboring-pixel charge sharing and the point-like quality of Bragg spot observations, which departs from previous-generation Bragg signals that were convoluted with a significant point-spread function.

In this article, we consider how a toolbox such as *cctbx* can contribute to the immediate data-processing events surrounding data collection and to the creation of improved algorithms to treat marginal data in general, including diffraction patterns exhibiting two or more lattices. After briefly describing the *cctbx* architecture and its potential to facilitate software collaboration (§2), we provide examples that illustrate its present (§3) and future (§4) uses in the beamline computing environment.

## 2. *cctbx* as a collaborative platform

**Figure 1:**
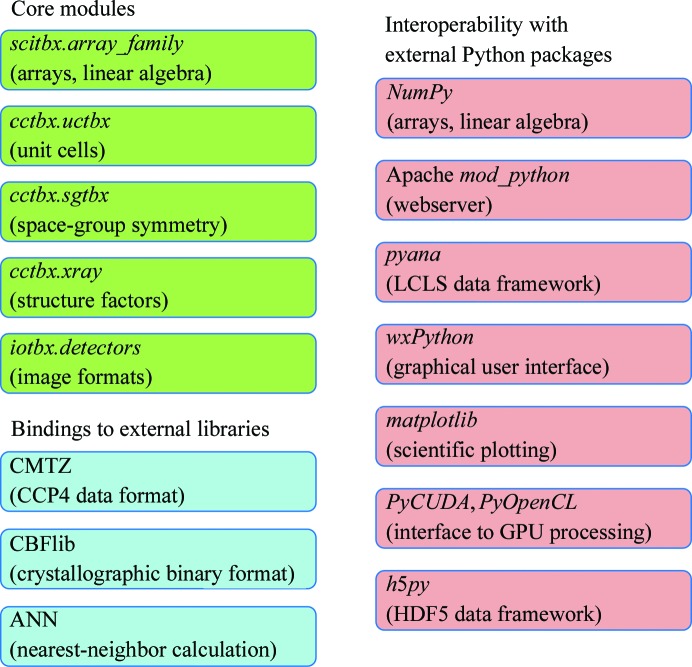
Overall organization of *cctbx*, showing selected modules relevant to the applications described in this article. In addition to standalone core modules, *cctbx* provides object-oriented Python bindings to the C-­language libraries CMTZ (Winn *et al.*, 2002 ▶), CBFlib (Bernstein & Ellis, 2005 ▶) and ANN (Arya *et al.*, 1998 ▶). Python scripting allows the *cctbx* code to interoperate with externally developed packages. Functions of interest are provided by the packages *NumPy* (http://www.numpy.org), *mod_python* (Trubetskoy, 2007 ▶), *pyana*, *wxPython* (Rappin & Dunn, 2006 ▶), *matplotlib* (http://matplotlib.org), *PyCUDA* (Klöckner *et al.*, 2012 ▶) and *h*5*py* (http://code.google.com/p/h5py).

As a long-term collaborative effort between several groups, *cctbx* has assumed the form of a warehouse of algorithms, libraries and tools (Fig. 1 ▶), from which the best one can be chosen to solve a given problem. Lower-level implementations impacting almost any crystallographic project include shareable memory-managed arrays, linear algebra, unit cells, space-group symmetry and structure factors. The *iotbx.detectors* package is of specific interest for data reduction, allowing data to be input from numerous X-ray detector file formats.

For programmers interested in rapidly testing new algorithms, *cctbx* thus offers the ability to efficiently express new ideas at a high level of abstraction. Consider the following four-line example, which at first appears to be a very simple segment of code in Python language,

image = ImageFactory(filename)

spots = image.get_spotfinder()

tiles = image.get_tile_manager()

graphics = image.get_flex_image()

Here, ImageFactory is a function that reads many types of detector data (with automatic detection of the format) and returns an image object that has a uniform interface. The interface includes functions to instantiate a spots object that can find and report the observed Bragg spots, a tiles object that contains geometry information allowing a data-reduction program to avoid signal integration on inactive areas of the image and a graphics object that allows the raw data to be rendered in an appropriate form within a graphical user interface. Thus, very complex applications can be written on top of simple commands.

Powerful interfaces like this are enabled by the hybrid-language *boost.python* architecture chosen for *cctbx* (Abrahams & Grosse-Kunstleve, 2003 ▶). Core functions are written in C++ and exposed as Python functions, resulting in excellent performance for algorithms that are CPU-limited when optimized with modern compilers. Furthermore, the interoperability of C++ and C makes it possible to link to indispensable libraries authored by third parties. These include the CCP4 CMTZ library for structure-factor input/output (Winn *et al.*, 2002 ▶), Herbert Bernstein’s CBFlib for crystallographic binary format (Bernstein & Ellis, 2005 ▶) and the University of Maryland Approximate Nearest Neighbor Library (Arya *et al.*, 1998 ▶), which is useful for matching up predicted and observed Bragg spot positions using a fast binary-tree algorithm. Meanwhile, higher-level concepts in the toolbox are expressed in Python scripting language, thus bringing together the compiled components of the program. New applications are generally prototyped in Python, with numerically intensive sections being ported to C++ as needed.

The source code in *cctbx* is comprised of contributions from numerous separately funded software projects such as *PHENIX* (Adams *et al.*, 2010 ▶), *LABELIT* (Sauter *et al.*, 2004 ▶), *OLEX*2 (Dolomanov *et al.*, 2009 ▶) and *xia*2 (Winter, 2010 ▶), which have all produced reusable core modules (Fig. 1 ▶) of general interest. Collaboration has been facilitated by hosting the code at the publicly accessible Sourceforge site (http://cctbx.sf.net), along with the use of a concurrent versioning system (Collins-Sussman *et al.*, 2008 ▶) that allows participants to document the purpose of each code revision.^1^ Nightly distributions are created automatically, allowing users to obtain the latest code in binary-executable form running on GNU/Linux, Mac OS X and Windows platforms. Furthermore, the nightly build process includes an extensive set of test scripts, run on all platforms, which verify that new code does not break the expected behavior of the existing code. Project contributors are expected to be diligent in writing these test cases to exercise any important feature, for it is this discipline that has allowed the project to accept contributions across continents for many years. Any failure of a test script shows up on a nightly web page, along with a stack trace identifying the point of failure, allowing corrective action.^2^

## 3. An extremely fast spotfinder for real-time applications

Python scripting gives the programmer access to numerous external code libraries, which have proven to be of enormous help in developing code that operates in a variety of experimental contexts. For the remainder of this article, we show how scripted tools originating outside the crystallographic domain (Fig. 1 ▶, right column) have been beneficially integrated with *cctbx*-based code, leading, for example, to the deployment of the *cctbx* Bragg spot picker for new experimental uses.

### 3.1. Synchrotron implementation

The *spotfinder* package within *cctbx* (Zhang *et al.*, 2006 ▶) selects candidate Bragg spots for autoindexing (Sauter *et al.*, 2004 ▶) and is therefore a fundamental component of synchrotron-based data processing. Bragg spot analysis has also become important in high-throughput sample screening, in which small partial data sets are streamed through automated pipelines such as *Web-Ice* (González *et al.*, 2008 ▶), *EDNA* (Incardona *et al.*, 2009 ▶) or *STARS* (Yamada *et al.*, 2008 ▶) that link spotfinding, autoindexing and strategy determination to select the best samples for full data collection. More recently, the introduction of microbeams has allowed specimens to be scanned with low-dose X-rays along a rasterized grid to optimize the sample-positioning step for crystals that are either difficult to see or inhomogeneous; for example, samples in lipidic cubic phase (Cherezov *et al.*, 2009 ▶). Analysis with *spotfinder* provides various measures of diffraction quality (number of spots, total spot intensity and resolution limit) and several beamline-control interfaces plot these statistics on a graphical display superimposed on the videomicrograph of the sample (Song *et al.*, 2007 ▶; Soltis *et al.*, 2008 ▶; Cherezov *et al.*, 2009 ▶; Hilgart *et al.*, 2011 ▶; Stepanov *et al.*, 2011 ▶; Bowler *et al.*, 2010 ▶; Aishima *et al.*, 2010 ▶; Winter & McAuley, 2011 ▶).

The overall speed of the raster scan depends on several experimental factors, including the granularity of the raster grid and the chosen exposure time, but it is clearly desirable that the spotfinding analysis should never be the rate-limiting step of the overall process. This became a software-engineering challenge after several beamlines deployed PILATUS 6M PAD hardware for raster scans with continuous sample motion and shutter-free data acquisition (Aishima *et al.*, 2010 ▶). Corresponding data rates reach 25 images s^−1^, yet the spotfinder procedure typically requires 2.0 s to sequentially read the data, classify the pixels as signal or noise, identify spots, eliminate ice or powder rings and apply spot-quality heuristics (Sauter, 2010 ▶). As none of these steps dominates the CPU usage, there is no easily identifiable portion of the code that can be rewritten to improve performance. Therefore, the most straightforward route to speed up overall throughput is to use multiple cores to process separate and independent images simultaneously.

**Figure 2:**
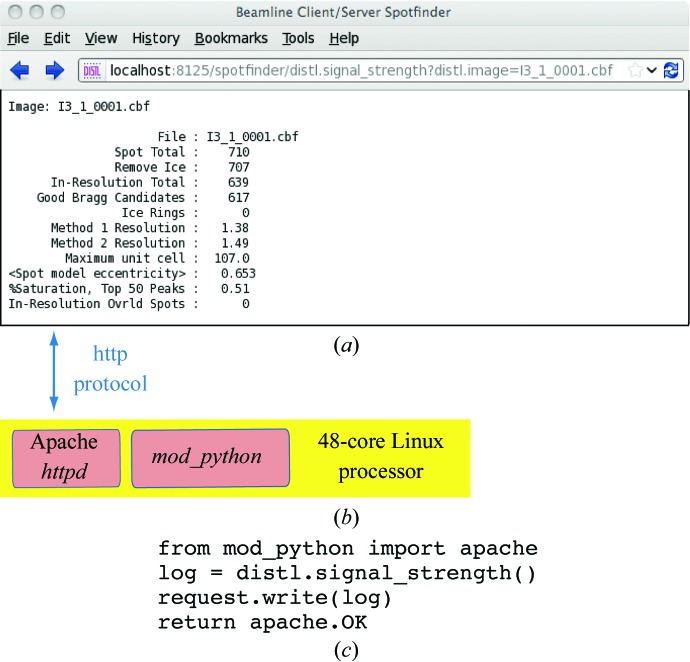
Client–server architecture for a high-throughput Bragg spot analyzer. The illustrated client (*a*) is a web browser, but the client is usually the beamline component responsible for the raster scan, such as *Blu-Ice* (McPhillips *et al.*, 2002 ▶) or *GDA* (Aishima *et al.*, 2010 ▶), implemented in any language that supports the HTTP protocol. The server (*b*) is a multicore Linux system running the Apache *httpd* daemon, which delegates incoming requests to one of 48 parallel child processes, each of which runs Python-language *cctbx* code mediated by the *mod_python* package (*c*). The server returns text-based output identical to that produced by the command-line program *distl.signal_strength*. There is also an option for the returned text to be formatted in extensible markup language (XML) suitable for automated control-system clients. Full instructions are given at http://cci.lbl.gov/labelit/html/client_server.html.

To implement concurrent processing, we chose a client–server paradigm in which the server maintains a persistent Unix process to analyze successive images, thus eliminating the need to reload the dynamic libraries (and saving 0.5 s per image). Rather than expending any effort writing new server code, readily available tools were selected. With minimal work (Fig. 2 ▶), the spotfinder service was refactored as a dynamic webpage served by the Apache program *httpd* (http://httpd.apache.org), which transparently handles multiprocessing by delegating each new image to the next available *httpd* child process. The child processes are configured to use *mod_python*, another freely available module that exposes a Python-language interpreter (Trubetskoy, 2007 ▶) suitable for running *cctbx* code within the Apache server. Within this paradigm, the client can be any beamline-control software component that conducts the raster scan, typically written by the synchrotron staff. A familiar application-layer protocol (HTTP) is used for client–server communication, allowing the server to be tested just by typing in a correctly formed address into a web browser (Fig. 2 ▶). As a general benchmark, it is possible to process PILATUS 6M images at a rate of 25 images s^−1^ on a 48-core 64-bit 2.2 GHz AMD Opteron machine running GNU/Linux (Sauter, 2011 ▶).

### 3.2. Free-electron laser implementation

A multiprocessing strategy is also effective in examining the data collected during serial femtosecond crystallography (SFX) experiments at X-ray free-electron lasers. Crystallo­graphic studies at these facilities (Chapman *et al.*, 2011 ▶) have focused firstly on the examination of crystal specimens that are too small (≤1 µm) for study at synchrotron sources (Johansson *et al.*, 2012 ▶; Koopmann *et al.*, 2012 ▶) and secondly on the characterization of metalloprotein catalytic centers (Kern *et al.*, 2012 ▶) that are susceptible to radiation damage on the exposure time scales required for data collection using synchrotron radiation (Yano *et al.*, 2005 ▶). Radiation damage is avoided by using femtosecond X-ray laser pulses to produce diffraction before damage processes have altered the structure (Lomb *et al.*, 2011 ▶; Barty *et al.*, 2012 ▶). Diffraction data collection differs dramatically from synchrotron-based protocols, chiefly owing to the short exposure time, which produces still shots rather than rotation data sets, and the high photon fluence, which destroys each sample after one shot, requiring the full data set to be built up from tens of thousands of stills from randomly oriented crystals (Kirian *et al.*, 2010 ▶, 2011 ▶; White *et al.*, 2012 ▶). The delivery of such a large number of crystals at a high rate has been accomplished by liquid-jet technology (DePonte *et al.*, 2008 ▶; Sierra *et al.*, 2012 ▶). While straightforward in principle, the combination of liquid-jet sample delivery with SFX data collection entails numerous variables that must be adjusted in real time, such as the relative positioning of the X-ray pulse and liquid jet, sample-flow parameters, beam attenuation, synchronization of beam and readout, and choice of microcrystal batch. To help fine-tune the experiment it is essential to have a rapid method for assessing the diffraction data quality, just as described above for the synchrotron-based raster scan.

**Figure 3:**
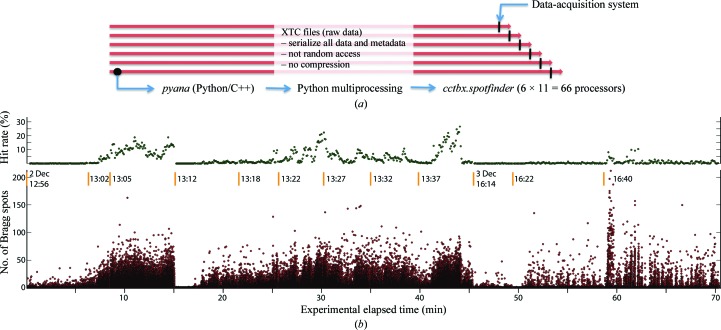
Concurrent processing of femtosecond crystallography data at LCLS with *cctbx.xfel*. A file-mediated approach is taken in which the data-acquisition system multiplexes the detector images to several serial-access binary streams written in extended tagged container (XTC) format (*a*). For data analysis, each of the six XTC files is assigned to a separate 12-core Linux node, on which the *pyana* framework reads the data within a single master process and delegates the analysis of consecutive images to as many as 11 child processes. *pyana* provides a Python-language callback hook to be executed once for each image, into which is inserted the *cctbx* spotfinder code. As the XTC file is on a shared-disk file system, data acquisition and processing are performed simultaneously. Although processing lags behind acquisition for any given XTC file, the ‘run’ is switched after a few minutes to a new XTC file, so the overall processing throughput roughly keeps up. (*b*) Bragg spot counts/image are shown for a 70 min 483 845-image thermolysin data set (Sierra *et al.*, 2012 ▶) broken into 12 runs starting at the indicated wall clock times. The hit rate (defined as the fraction of images with ≥16 Bragg spots within a defined area) is plotted over a 5 s sliding window. The total number of hits is 15 094.

The *cctbx.spotfinder* package was therefore extended to process diffraction from the Coherent X-ray Imaging instrument (Boutet & Williams, 2010 ▶) at the LCLS, with images acquired at 120 Hz using a Cornell-SLAC PAD detector (Philipp *et al.*, 2010 ▶). While the native data-acquisition (DAQ) environment at this beamline can display images, the framing rate is too high for per-frame visual inspection, so the DAQ display is configured to sample only one tenth of the images collected and to display a 1 s cumulative picture over many frames. In parallel, the new software (designated *cctbx.xfel*) separately quantifies every image from the full data stream and displays summary statistics (Fig. 3 ▶) such as the number of strong Bragg spots on each image and the hit rate (the number of images that exceed a cutoff count during a sliding time window). To implement the necessary multiprocessing and access the data stream, *cctbx.xfel* interacts with *pyana*, a Python/C++ data-processing framework developed by the SLAC Photon Controls and Data Systems group. This arrangement allows near-real-time Bragg spot analysis (Fig. 3 ▶) by utilizing 100–150 cores on 64-bit 2.5 GHz Intel Xeon processors.

## 4. Adapting the Python toolset for challenging diffraction patterns

The high data-throughput rate is not the only outstanding issue arising from the adoption of PAD-type detectors. The nature of the data is also changing. Now that raster scanning is readily implemented, one can anticipate data sets that probe many positions within the sample loop, with some positions producing high-quality diffraction spots and other positions generating only marginal data. Similar comments can be made about SFX protocols, in which the ensemble of diffraction patterns is found to represent a spectrum of crystal qualities and limiting resolutions.

**Figure 4:**
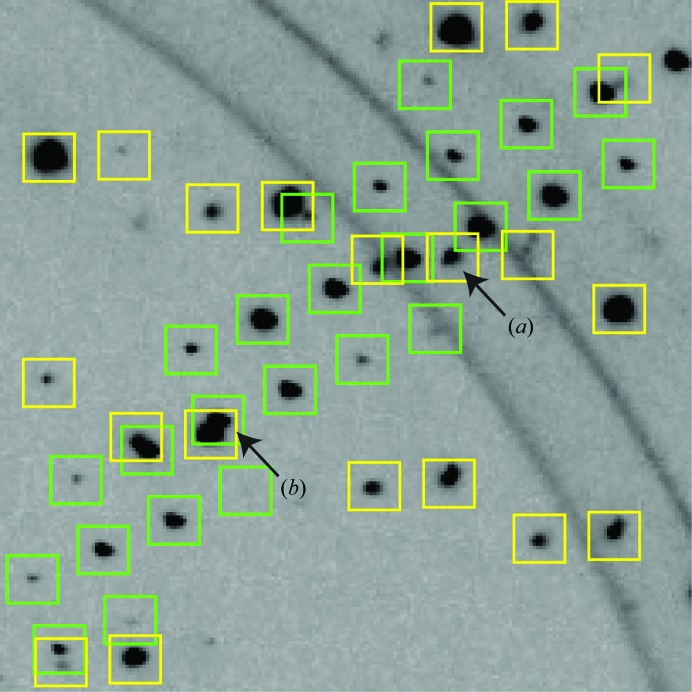
Indexing model from an exposure illuminating two lysozyme microcrystals collected at ALS beamline 5.0.1 using an ADSC Q315 detector. Most reflections on the two lattices (yellow and green) are well separated, but some come close enough to impinge on the integration box chosen for modeling spots on the other lattice (*a*), while a few overlap outright (*b*).

As a specific example, consider the likelihood that numerous data sets will reveal multiple lattices, since loop scanning and liquid-jet sample delivery are expected to produce some shots with more than one crystal in the beam. Such a diffraction pattern from a recent synchrotron data set is shown in Fig. 4 ▶. Multi-crystal indexing is now possible (Sauter & Poon, 2010 ▶; Paithankar *et al.*, 2011 ▶; Sørensen *et al.*, 2012 ▶; Powell *et al.*, 2013 ▶) and data integration is straightforward with standard programs, if one assumes that the Bragg spots do not overlap. However, if one is to take proper account of the fraction of spots that do actually overlap or lie close then new integration methods are needed. Various approaches have been discussed, ranging from the exclusion of overlapping spots after the data have been integrated (Buts *et al.*, 2004 ▶; Paithankar *et al.*, 2011 ▶) to the deconvolution of overlapping spots at the time of integration (Bourgeois *et al.*, 1998 ▶; Schreurs *et al.*, 2010 ▶).

The approach of Schreurs *et al.* (2010 ▶), in particular, proposes the use of fine-grained models that interpret the varying size and shape of Bragg spots on the image as arising from physical properties of the experiment, such as beam bandwidth and divergence, along with crystal size and mosaicity. This strategy is promising not only for interpreting diffraction from multiple lattices, but also in other cases where the spot shape is extended or diffuse (Nave, 1998 ▶; Tsai *et al.*, 2009 ▶). It is likely that experimentation will be required to arrive at the best model for any given data set. In support of such work, Python scripting offers an easily adaptable framework for experimentation, and two useful developments are discussed in this section.

### 4.1. A graphics toolbox for new data-reduction methods

**Figure 5:**
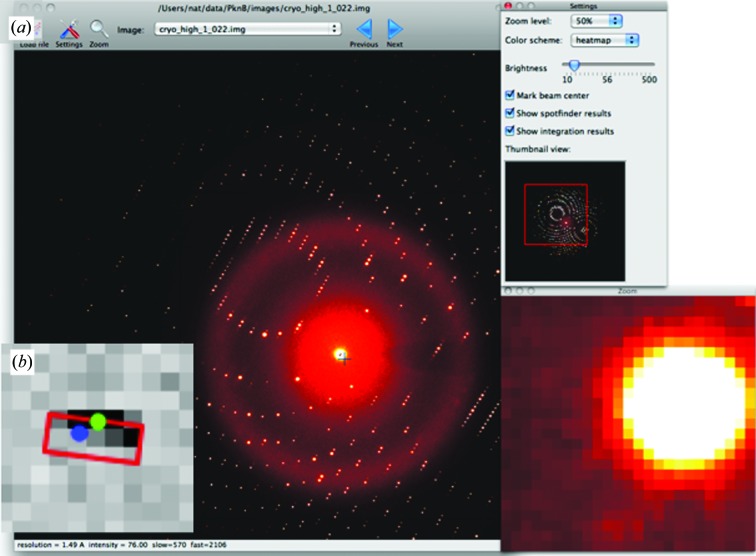
(*a*) Cross-platform *wxPython*-based *phenix.image_viewer* application included in *cctbx*. (*b*) Detail of the prototype *cctbx.image_viewer*, which exposes a programming interface for displaying models. Here, the red box and blue dot are *alternate models* of the Bragg diffraction recorded on a PAD detector; the models have not been optimized and thus differ substantially from the center position of the observed Bragg spot (green dot).

A Python-based image viewer for diffraction data has recently been added to *cctbx* (Fig. 5 ▶ *a*). This graphical user interface (GUI), which relies on the cross-platform *wxPython* toolkit (Rappin & Dunn, 2006 ▶), was inspired by other data viewers such as *adxv* (Szebenyi *et al.*, 1997 ▶), but is amenable to subclassing to support new algorithm development. The original publication (Echols *et al.*, 2012 ▶) described the program (*phenix.image_viewer*) as being included in the *PHENIX* package; however, it is now also available under the open-source *cctbx* license in both source-code and binary forms.

Present efforts to extend data-reduction methods will rely heavily on viewing the measured data compared with various models, thus prompting the emphasis on developing a flexible GUI. New code currently being prototyped (*cctbx.image_viewer*) permits easy navigation through the data using mouse click-and-drag motions to pan the image and mouse scroll-wheel motions to zoom in and out, similar to the actions of the popular web service Google Maps. An arbitrary number of colored overlays can be added to the image, for example quadrilaterals and dots to represent various physical models of the diffraction, aligned with the data image to subpixel precision (Fig. 5 ▶ *b*). Provision has been made to map the pixel coordinates of the detector onto the laboratory coordinate system, making it possible to represent PAD detectors composed of numerous silicon tiles that may have relative tilt and fractional pixel displacements. This same facility can be readily adapted to cylindrical or spherical detectors. While the mapping from detector to laboratory space consumes extra CPU cycles, the response time is minimized by rendering only the portion of the data that is currently being viewed at the present zoom level, while caching ahead the neighboring tiles to anticipate mouse-driven pans. The infrastructure for these features, based on *wxPython*, was derived from the pySlip project authored by Ross Wilson (http://code.google.com/p/pyslip).

### 4.2. Python-mediated GPU computing

Graphics-processing units (GPUs) are a low-cost avenue for accelerating commodity hardware for high-performance computing and are extensively used in the computational sciences (Hwu, 2011 ▶), yet only a few crystallographic applications have been reported (Favre-Nicolin *et al.*, 2011 ▶; Schnieders *et al.*, 2011 ▶).

However, massively parallel GPU architecture has great potential to assist in data reduction in two ways: either by speeding up the workflow to keep pace with data acquisition or by allowing the testing of more detailed models. GPU computing power raises the possibility of treating data reduction as an optimization problem (Schreurs *et al.*, 2010 ▶), in which the structure factors are treated as unknowns to be modeled along with the experimental parameters (bandwidth, divergence, crystal size and mosaicity, as mentioned above), all of which are adjusted to create the best pixel-by-pixel fit between model and observation.

**Figure 6:**
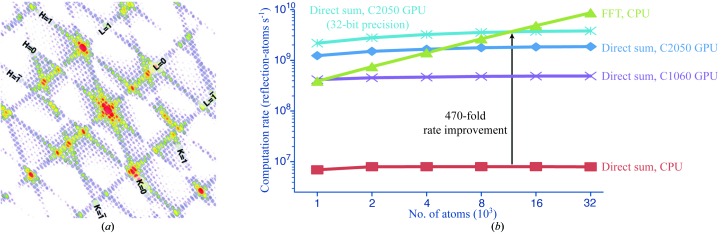
(*a*) Low-order fringe pattern for a photosystem I crystallite calculated on a GPU and similar to that actually observed at the LCLS (Chapman *et al.*, 2011 ▶). (*b*) Computational efficiency of evaluating (1) scaling as *N* ^2^ (number of atoms × number of structure factors). The CPU calculation was performed single-threaded on a 64-bit Intel Xeon (2.4 GHz), 8 MB cache, 23.5 GB RAM running Scientific Linux 5.4 with code compiled under GCC 4.4.2. GPU calculations were either on an Nvidia C1060 (Tesla, 1.30 GHz), 4.0 GB on-device memory, 960 hardware cores or on the higher-performance Nvidia C2050 (Fermi, 1.15 GHz), 2.6 GB on-device memory, 448 hardware cores; both were programmed in CUDA. The top plot (blue crosses) depicts calculations run with 32-bit (single) precision; otherwise, calculations were in 64-bit (double) precision. A comparison is given with the FFT method, which scales as *N*log*N*. The loss of accuracy observed on moving from 64-bit to 32-bit precision is generally less than the loss of accuracy (typically 0.8%) resulting from use of the FFT approximation rather than (1). Example code is available at http://cctbx.svn.sourceforge.net/viewvc/cctbx/trunk/cctbx/x-ray/structure_factors/from_scatterers_direct_parallel.py. Python bindings for CUDA utilize the *PyCUDA* package (Klöckner *et al.*, 2012 ▶). Benchmarks in (*b*) are performed on a single unit cell in space group *P*1, while the simulation in (*a*) is over all atoms in 10 × 12 × 14 unit cells in space group *P*6_3_. Simulation (*a*) scales as *N* ^2^ as it uses (1).

In view of this, we examine the use of GPUs to calculate structure factors from atomic coordinates using direct summation (Favre-Nicolin *et al.*, 2011 ▶), for the direct summation approach gives a computational avenue for modeling Bragg spots that are extended in size and shape owing to crystal imperfections. The simulated pattern in Fig. 6 ▶(*a*) is one such example, in which a fringe function arises from the small number of unit cells along each crystal axis; this type of pattern was observed by Chapman *et al.* (2011 ▶) for crystallites of photosystem I. Another phenomenon that may be amenable to such modeling is lattice-translocation disorder, in which successive crystal layers are randomly displaced, producing Bragg spots that are streaked along one axis (Tsai *et al.*, 2009 ▶).

While porting an application to a GPU platform can certainly be beneficial (Fig. 6 ▶ *b*), there are also limitations, which can be appreciated by considering in detail how the direct summation is implemented. The macromolecular structure factor *F*_*H*_ is given by
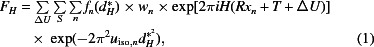
where *H* is the Miller index, 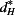 is its associated reciprocal-lattice spacing, *f_n_* is the atomic form factor of the *n*th scatterer with fractional coordinates *x*_*n*_, *u*_iso,*n*_ is the isotropic displacement parameter, *w_n_* the occupancy factor, *R* and *T* are the rotation and translation parts, respectively, of the symmetry operations *S* of the space group, and the operators Δ*U* span all unit cells in the crystal. While it is typically more efficient in macromolecular crystallography to estimate *F_H_* by fast Fourier transformation of the electron density, the direct summation in (1) is the appropriate expression for computing diffraction at fractional values of the Miller indices in crystallites with a small number of unit cells or cell-to-cell disorder. (1) reduces to the continuum scattering expression when there is just one unit cell. We implemented (1) on Nvidia GPUs containing either 448 or 960 hardware cores (Fig. 6 ▶). Our version differs from the previous implementation (Favre-Nicolin *et al.*, 2011 ▶) in allowing symmetry operations and isotropic displacement parameters. It is also tightly integrated with native *cctbx* data types, permitting a simple Python script to read in a PDB file with the standard *cctbx* toolset and to then immediately calculate direct-summation structure factors on attached GPU hardware. The kernel code that executes on the GPU is <150 lines written in Nvidia’s CUDA language, which is similar to C.

The calculation is organized such that each *F_H_* is evaluated by a separate thread.^3^ In contrast to the situation for general-purpose CPUs, which automatically use the on-die cache to speed up data access, the GPU interface places responsibility for data transfer directly on the programmer. Data transferred from the CPU host to the GPU device can have two initial destinations. Firstly, there is a small block (64 KB) of constant memory that is rapidly readable by the GPU threads, which is useful in our case for atomic form factor Gaussian coefficients and symmetry operators *S*. Secondly, there is ample global memory (3 GB) to store all the fractional atomic coordinates *x_n_*, along with the output list of *F_H_* prior to its return transfer to the host. The GPU parallelizes its work with a single-instruction multiple-thread model (Kirk & Hwu, 2010 ▶) in which blocks of 32 threads execute instructions in lockstep. Thread blocks have access to only a tiny amount (48 KB) of on-die shared memory; this poses a memory-management challenge since the atomic coordinates must ultimately be transferred on-die for the calculation. We make this efficient by having the 32-thread blocks coalesce. Each thread reads coordinates for a single atom; thus, 32 atomic coordinates are read simultaneously by synchronized threads and each data element can be used by each of the 32 threads before it is replaced in the next data-transfer cycle. By minimizing the number of global-to-shared memory transfers in this way, we are able to simulate a fringe pattern for a 10 × 12 × 14 unit-­cell crystallite of photosystem I in under 2 min (Fig. 6 ▶ *a*). The calculation is 200-fold faster than the equivalent double-precision performance on a single-process CPU (Fig. 6 ▶ *b*). 

This short description shows that algorithms must inevitably be refactored to make optimal use of the hardware resources of the GPU. Owing to this extra effort, it is only beneficial to focus on small sections of the problem (such as the structure-factor formula) that are truly rate-limiting, while performing the balance of the calculation on the CPU. Furthermore, it is critical to choose the correct programming pattern for parallelizing the algorithm (Owens *et al.*, 2007 ▶). In our example, each thread was chosen to represent one structure factor and we were able to use data-transfer coalescence to efficiently gather all the atomic coordinate inputs into each thread. The alternate choice, which would be unproductive, is to use threads to represent the contribution of individual atoms. In this pattern, each thread scatters its numerical results across all of the output channels (the structure factors); however, this is extremely inefficient because a global lock must be placed around the output variable each time a thread adds its contribution. While we have not yet used GPUs for routine diffraction data reduction, it is interesting to speculate how these lessons would apply to data modeling. For example, when emulating recently described ray-tracing approaches (Diederichs, 2009 ▶; Schreurs *et al.*, 2010 ▶) we may benefit from mapping threads to individual detector pixels, which would gather the ray-tracing contributions from an ensemble of input optical rays.

## 5. Availability

The software described here is available in source and binary distributions at http://cctbx.sf.net under a permissive BSD-like open-source license that allows unrestricted academic or commercial reuse. Contributions of new code can be arranged with the authors. Where possible, the binary downloads have been bundled with the supporting packages described in the text.

## Footnotes

1 Prospective contributors may contact the authors to obtain developer credentials for the project website.

2
http://cci.lbl.gov/cctbx_build/. Navigate to the nightly logs by selecting ‘Show unreleased builds’ and then ‘summary’.

3 The kernel, encoding the computation for one thread, calculates a single *F_H_*.
